# Risk factors for compensatory hyperhidrosis following CT-guided percutaneous radiofrequency sympathectomy: A retrospective observational study

**DOI:** 10.1097/MD.0000000000048976

**Published:** 2026-05-22

**Authors:** Chaobo Ni, Zixin Han, Bohan Hua, Huadong Ni, Ming Yao

**Affiliations:** a Zhejiang Chinese Medical University, Hangzhou, Zhejiang, China; b Department of Anesthesiology and Pain Research Center, The Affiliated Hospital of Jiaxing University, Jiaxing, Zhejiang, China.

**Keywords:** compensatory hyperhidrosis, dynamic nomogram, radiofrequency sympathectomy, risk factor

## Abstract

Compensatory hyperhidrosis (CH) is a significant complication following surgery for primary hyperhidrosis, impairing patients’ postoperative experience and quality of life. Radiofrequency sympathectomy (RFS) is one of the main minimally invasive surgeries for hyperhidrosis, yet high-risk factors and patient experiences of postoperative CH are rarely reported. This retrospective observational study (Strengthening the Reporting of Observational Studies in Epidemiology compliant) aimed to identify significant risk factors for CH after RFS and to develop a dynamic nomogram for individualized risk prediction. A total of 410 primary hyperhidrosis patients who underwent RFS between January 2018 and March 2023 were enrolled. A CH database was established via systematic follow-up to track postoperative sweating patterns. Detailed clinical assessments included the Numeric Rating Scale (NRS), Hyperhidrosis Disease Severity Scale (HDSS), and Hospital Anxiety and Depression Scale (HADS). Advanced statistical techniques, including logistic regression analysis, were used to identify potential risk factors for CH. Of the 410 patients, 281 (68.5%) developed CH, with 105 (25.6%) reporting moderate-to-severe symptoms. Multivariate analysis revealed 4 significant predictors of CH: male gender (odds ratio [OR] = 1.69); higher NRS scores (OR = 1.16); lower immediate postoperative HDSS (OR = 0.48); higher preoperative HADS scores (OR = 1.11). A dynamic, web-based nomogram was also developed to provide personalized CH risk prediction. Male gender, higher intraoperative NRS scores, lower immediate postoperative HDSS, and higher preoperative HADS scores were identified as independent risk factors. The constructed dynamic nomogram enables individualized risk prediction and supports clinicians in providing personalized preoperative counseling and perioperative management for patients undergoing RFS.

## 1. Introduction

Primary hyperhidrosis (PH) is a chronic disorder characterized by excessive sweating beyond normal thermoregulation of the body.^[1]^ Percutaneous radiofrequency thoracic sympathectomy, a new minimally invasive modality, has been demonstrated to be a potentially viable and safe treatment for palmar hyperhidrosis.^[2]^

Radiofrequency sympathectomy (RFS) physically destroys the corresponding sympathetic nerves, thereby blocking electrical signals that reach the effectors.^[3]^ The incidence of compensatory hyperhidrosis (CH) after computed tomography (CT)-guided RFS for PH has been reported to be in 15 to 84%.^[4,5]^ This may be owing to differences in the surgical indications, surgical efficacy, criteria for determining CH, and follow-up time. CH manifests as increased sweating in areas of the body other than the primary site, often involving the back, abdomen, groin, and thigh, leading to considerable discomfort.^[6]^ However, the etiology of CH remains unclear. It has been regarded as a thermoregulatory response in the past, wherein the overall sweating amount remains constant, and sweating is transferred from the sympathetic denervation area to the non-sympathetic denervation area. Some scholars also believe that the reflex sweating is caused by the abnormal regulatory reflex between the sympathetic nerve and the hypothalamic sweating regulatory center, that is, the negative feedback is cut off, resulting in a non-sympathetic area receiving infinite positive feedback.^[7]^

Although some researchers have explored the use of sequential thoracoscopic sympathectomy to reduce CH following sympathetic nerve electrocautery, the evidence remains limited and inconclusive.^[8]^ Many existing studies are small-scale and lack standardized outcome measures, underscoring the need for more robust, large-scale research to establish effective strategies for preventing and managing CH. Therefore, the prevention, diagnosis, and treatment of CH remain in the exploration and experimental stages. Our research group was committed to exploring the efficacy of RFS in the treatment of PH in the early stage, and found the serious problem of CH in this process.^[9]^

Therefore, this retrospective observational study aimed to identify significant risk factors for CH after RFS and to develop a dynamic nomogram for individualized risk prediction.

## 2. Material and methods

### 2.1. Study population and design

This retrospective observational study was approved by the Ethics Committee of the Affiliated Hospital of Jiaxing University, with a waiver of informed consent (approval number: 2022-KY-093). Eligible participants were patients diagnosed with PH who underwent CT-guided percutaneous RFS at our institution during the study period (January 2018–March 2023), all of whom met the established diagnostic criteria for PH.^[10]^

Inclusion criteria were defined as follows: conformed to the internationally validated diagnostic criteria for PH^[10]^; received elective CT-guided percutaneous sympathetic nerve intervention (chemical neurolytic block or radiofrequency thermocoagulation sympathectomy); completed the full scheduled postoperative follow-up, with intact data for postoperative sweating pattern tracking.

Exclusion criteria were defined as follows: comorbidities potentially inducing sweating symptoms (e.g., hyperthyroidism, menopausal syndrome, diabetes mellitus); inability to comprehend assessment questions or refusal to complete follow-up; and missing critical clinical data.

**Figure 1.**
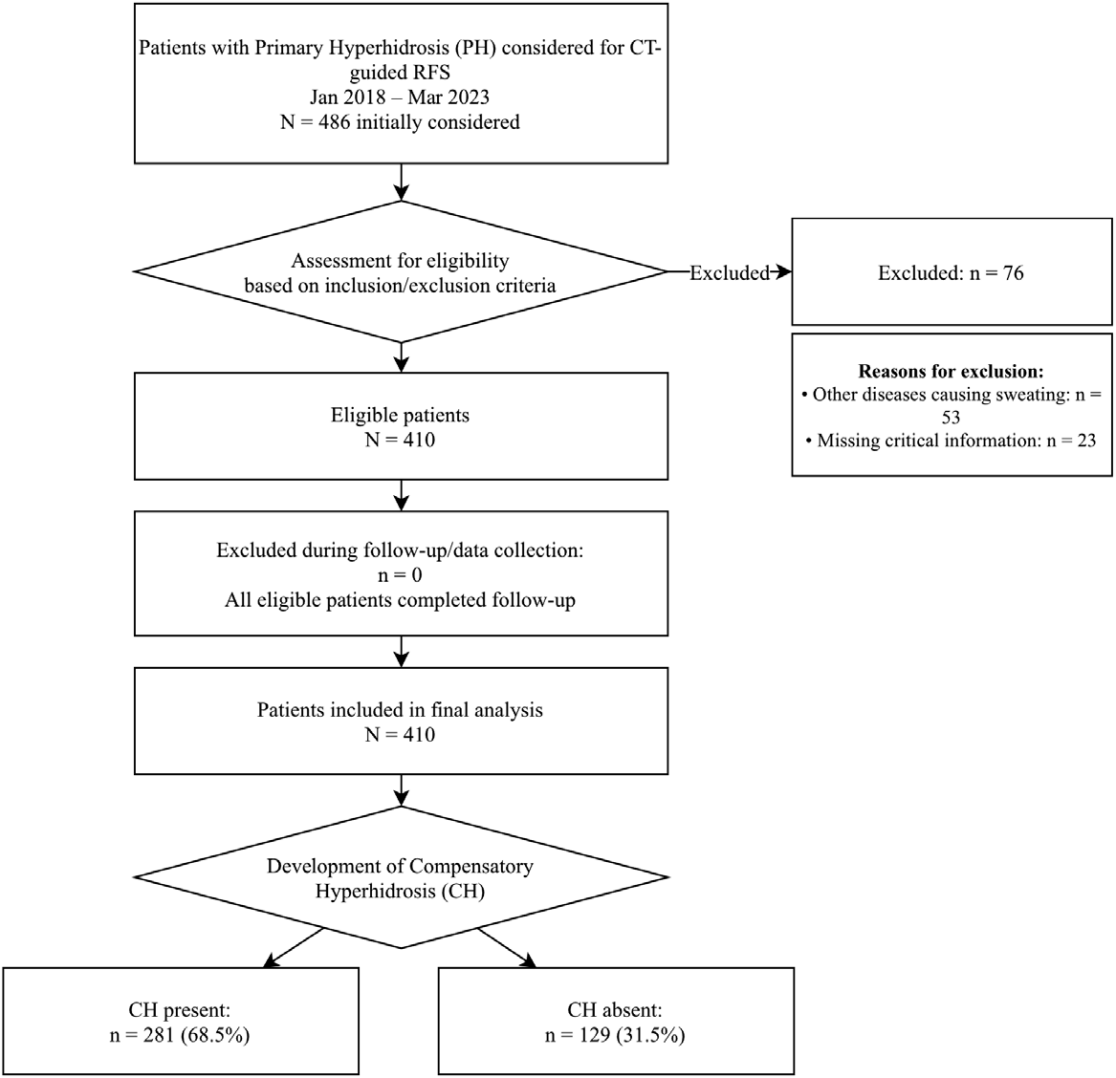
Patient Flowchart of Cohort Selection for CH Following CT-Guided RFS in PH Patients. CH = compensatory hyperhidrosis, CT = computed tomography, N/n = number of patients, PH = Primary hyperhidrosis, RFS = radiofrequency sympathectomy.

The cohort selection process is illustrated in Figure 1 (Patient Flowchart): a total of 486 patients scheduled for CT-guided RFS were initially screened, among whom 76 were excluded due to secondary hyperhidrosis (n = 53) or missing critical clinical data (n = 23). This yielded 410 eligible patients, all of whom completed the scheduled follow-up with no loss to follow-up and were therefore included in the final statistical analysis. These participants were further stratified into 2 subgroups based on the development of CH: 281 patients (68.5%) with CH and 129 patients (31.5%) without CH.

### 2.2. Sample size calculation

Given the retrospective observational design, no formal prospective sample size calculation was performed prior to data collection, and the sample size (n = 410) was determined by all eligible patients who underwent CT-guided RFS for PH at our institution between January 2018 and March 2023, consistent with common practice for retrospective studies using the entire available cohort within a defined timeframe; however, post hoc power analysis confirmed statistical reliability for multivariable logistic regression: based on an observed CH incidence of 68.5% (281/410) and the rule of thumb of at least 10 events per predictor variable, the 281 CH events could accommodate up to 28 predictor variables, well exceeding the 4 retained in the final model, and our cohort of 410 patients is one of the largest single-center studies on CH following RFS compared with previous similar studies, enhancing the generalizability and statistical power of the findings.^[11]^

### 2.3. Data collection

The primary end point was CH. CH was defined as increased sweating in parts of the body other than the primary site, and the severity of sweating and patient tolerance were assessed using the hyperhidrosis disease severity scale (HDSS), with postoperative HDSS > preoperative HDSS for other sites; 4 levels of the HDSS were as follows:^[12]^

Score 1: My sweating is never noticeable and never interferes with my daily activities.

Score 2: My sweating is tolerable but sometimes interferes with my daily activities.

Score 3: My sweating is barely tolerable and frequently interferes with my daily activities.

Score 4: My sweating is intolerable and always interferes with my daily activities.

The secondary outcomes included the following: Demographic information and surgical parameters were collected from the medical records, including sex, age, disease duration, inducement, primary site, surgical segment, perfusion index, skin temperature of hyperhidrotic areas (T), numeric rating scale (NRS), dermatology life quality index (DLQI) and pre-hospital anxiety and depression scale (HADS). Telephonic follow-ups were regularly conducted to create the database.

NRS was used to assess the intraoperative pain level. 0 was classiffed as no pain, 1 to 3 was classiffed as mild pain, 4 to 6 was classiffed as moderate pain, and 7 to 10 was classiffed as severe pain. Patients chose the value that matched their own pain level from 0 to 10.

The DLQI is a validated instrument designed to evaluate the impact of dermatological conditions on patients’ quality of life. This standardized questionnaire comprises 10 items assessing 6 domains: symptoms and feelings, daily activities, leisure, work/school, personal relationships, and treatment effects. Each item presents 4 response options scored from 0 (no impact) to 3 (severe impact), with total scores ranging from 0 to 30. Higher composite scores indicate greater impairment of health-related quality of life, where 0 to 1 represents no effect, 2 to 5 small effect, 6 to 10 moderate effect, 11 to 20 very large effect, and 21 to 30 extremely large effect on quality of life. The DLQI demonstrates good reliability (Cronbach *α* > 0.90) and has been widely adopted in both clinical practice and research for objective quantification of dermatological disease burden.

The pre-HADS was divided into an anxiety subscale (A) and a depression subscale (D). There were 14 items, each scored on a scale of 0 to 3. The higher the score, the more likely the presence of anxiety and depression.

### 2.4. Surgery

The procedure, efficacy, and possible risks were explained to the patients in detail, and surgical informed consent was obtained. Fasting was performed for 6 hours, and intravenous access was established for each patient before the procedure. The heart rate, noninvasive blood pressure, oxygen saturation, terminal skin temperature, and perfusion index were routinely monitored. The surgeries were performed by 2 physicians with more than 5 years of experience in neuromodulation. All procedures were performed under CT guidance.

Lumbar sympathetic radiofrequency: A 22-G blunt-tipped radiofrequency needle (Inomed Health Ltd.) was inserted through the paravertebral space to the anterolateral aspect of the vertebral body; the needle core was withdrawn, and the supporting electrode was inserted along the cannula. The position of the needle tip was adjusted until the resistance of the tissue around the tip of the test electrode was between 250 and 550 Ω. The sensory and motor electrical stimulation tests were negative, and after confirming that the position of the radiofrequency needle tip was correct, the temperature was set to 90°C. Thermocoagulation lasted for 60 seconds and was repeated for 3 cycles.^[13]^

Thoracic sympathetic radiofrequency: The positioning method and radiofrequency parameters were the same as previously described. The target was the upper edge of the rib head. No limb numbness or activity disorder was observed. CT revealed no pneumothorax in the lung window. The radiofrequency electrode was removed at the end of the treatment. After removing the needle, the puncture point was attached locally using a bandage. Patients returned to the ward if their vital signs stabilized.^[11]^

The surgical segments were determined based on the location of the patients’ PH. Specifically, thoracic sympathetic radiofrequency was mainly performed at T3 (n = 89), T4 (n = 276), and T5 (n = 33); lumbar sympathetic radiofrequency was primarily targeted at the L3 segment (n = 12). All segment localizations were confirmed via CT guidance using anatomical landmarks (costal head/anterior vertebral body edge) and were documented in detail in the surgical records.

### 2.5. Statistical analysis

All statistical analyses were performed using the R (version 4.1.2; R Foundation for Statistical Computing, Vienna, Austria) software. Shapiro–Wilk test was used to analyze whether the data followed a normal distribution, which is expressed as mean ± standard deviation ($\bar{X}\pm s$) for normally distributed data and median (interquartile spacing) for non-normally distributed data. The paired *t*-test was employed to compare normally distributed data between the 2 groups. The Wilcoxon rank sum test was used for non-normally distributed data, and the chi-square test was used to compare differences in count data. Statistical significance was set at *P* < .05.

Initially, univariate analysis was used to identify variables that may affect the occurrence of CH, and relevant variables (*P* < .05) were included in multivariate logistic regression analysis to determine the independent predictors of CH and construct a dynamic nomogram for predicting CH. Internal validation was performed using 100 bootstrap samples to calculate the adjusted concordance index, and a calibration curve was constructed to assess the model performance. Statistical significance was prospectively set at *P* < .05. All *P* values are reported without a leading zero (e.g., *P* = .022) and as actual values rather than thresholds (e.g., *P* = .712 instead of *P* > .05).

### 2.6. Bias mitigation

To address potential biases inherent to the retrospective observational design, we implemented targeted measures: recall bias was minimized by prioritizing objective data extraction from electronic medical records and using standardized scales (HDSS, NRS, HADS) for structured telephone follow-ups; selection bias was reduced via strict predefined inclusion/exclusion criteria and consecutive enrollment of all eligible patients during the study period; measurement bias was controlled through consistent use of validated assessment tools and standardized surgical procedures (performed by experienced physicians with uniform CT-guided parameters); confounding bias was adjusted for via hierarchical statistical analyses (univariate and multivariate logistic regression) and stratified analysis of surgical segments; internal validity was enhanced by adhering to Strengthening the Reporting of Observational Studies in Epidemiology guidelines, clinical trial registration, ethical approval, and bootstrap validation of the predictive model; additionally, a dedicated CH database with cross-verified data entry mitigated documentation errors, collectively improving the credibility and generalizability of the findings.

## 3. Results

### 3.1. Demographic characteristics

A total of 410 eligible PH patients were finally enrolled in this study, and were stratified into the CH group (n = 281) and non-CH group (n = 129) according to the occurrence of postoperative CH. There was a statistically significant difference in gender distribution between the 2 groups (*P* = .043), while no significant differences were found in other baseline demographic and clinical characteristics (all *P* > .05, Table 1). Most patients presented with palmar or plantar hyperhidrosis, with a median disease course of 17 months (interquartile range: 12–23). More than 75% of patients had no obvious inducement for sweating attacks, and all patients had moderate-to-severePH with significant impairment of quality of life (Table 2).

**Table 1:** Demographic data of all enrolled patients.

Parameter	Level	Total	CH (n = 281)	Non-CH (n = 129)		*P* value
Gender	Male	205 (50%)	150 (53.4%)		55 (42.6%)	.043
	Female	205 (50%)	131 (46.6%)	74 (57.4%)		
Age	Age of onset	6 (6–12)	6 (6–12)	6 (6–12)		.832
	Age of modulation	25 (21–31)	25 (21–31)	25 (21–31)		.408
Smoking	No	346 (84.4%)	231 (82.2%)	115 (89.1%)		.072
	Yes	64 (15.6%)	50 (17.8%)	14 (10.9%)		
BMI		21.78 (19.58–24.24)	21.78 (19.58–24.24)	21.74 (19.56–24.23)		.094
Family history	No	278 (67.8%)	186 (66.2%)	92 (71.3%)		.302
	Yes	132 (32.2%)	95 (33.8%)	37 (28.7%)		

BMI = body mass index, CH = compensatory hyperhidrosis, mo = months, n = number of patients.

**Table 2:** Preoperative experiences of patients.

Course of disease (mo)	Median	(1st, 3rd)
	17	(12,23)
Sweating area (%)	Median	(1st,3rd)
	11	(6,12)
Primary site	No	%
Hand	322	78.5
Foot	259	63.2
Head	86	21.0
Face	85	20.7
Neck	3	0.7
Axilla	25	6.1
Chest	5	1.2
Back	5	1.2
Forearm	2	0.5
Upper-arm	2	0.5
Thigh	2	0.5
Leg	2	0.5
Hip	1	0.2
Inducement	No	%
None	316	77.1
Yes	94	22.9
Preoperative HDSS	No	%
3	89	21.7
4	321	78.3
DLQI	No	%
0–1	0	0.0
2–5	8	2.0
6–10	151	36.8
11–20	251	61.2
21–30	0	0.0

DLQI = dermatology life quality index, HDSS = hyperhidrosis disease severity scale.

### 3.2. Patient experiences of CH

**Figure 2.**
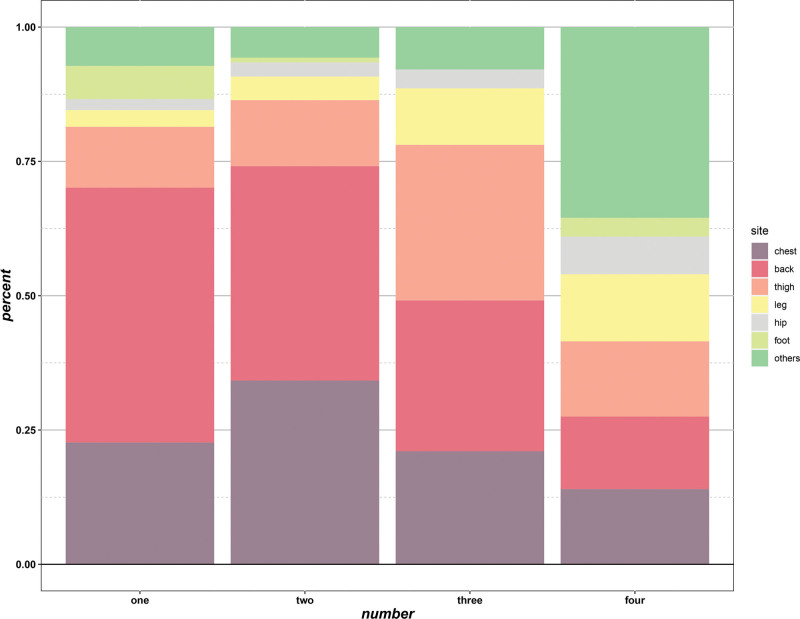
The regional distribution of CH by number of areas involved. CH = compensatory hyperhidrosis.

**Figure 3.**
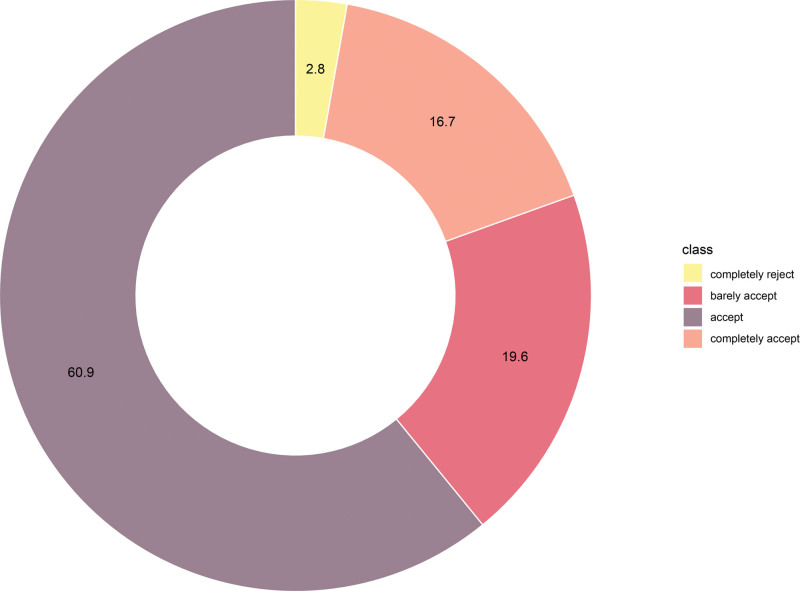
Patient acceptance of CH in 4 categories. CH = compensatory hyperhidrosis.

**Figure 4.**
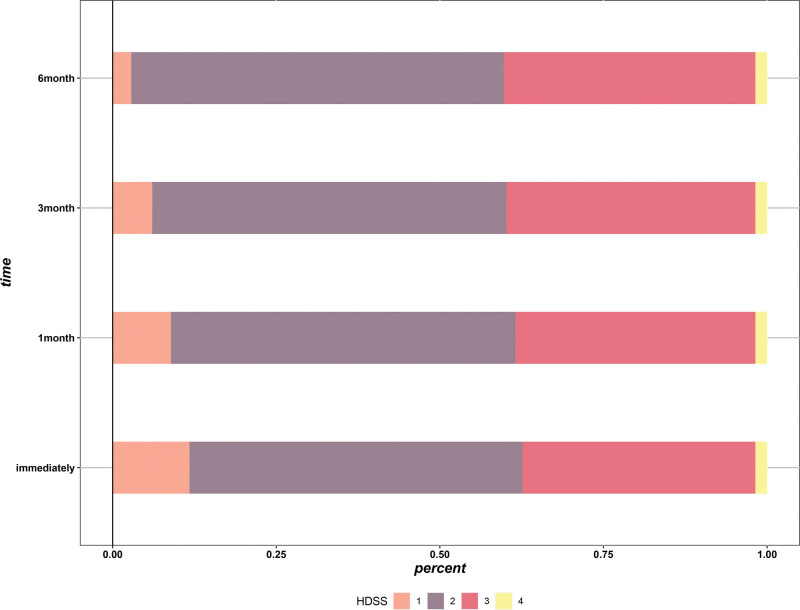
Changes in severity of CH over time. CH = compensatory hyperhidrosis, HDSS =

The overall incidence of CH was 68.5% in the cohort, with moderate-to-severe symptoms reported in a quarter of patients. CH most commonly involved the chest, back and lower extremities (Fig. 2). The vast majority of patients (60.9%) reported acceptable tolerance to CH, with only a small proportion of patients (2.8%) reporting complete intolerance (Fig. 3). The severity of CH remained stable within 6 months after surgery, with no significant temporal change in HDSS scores (Fig. 4), indicating that CH mainly occurs in the immediate postoperative period.

### 3.3. Risk factors for CH

Univariate logistic regression analysis was performed to screen variables potentially associated with CH occurrence, and 6 variables were identified with statistical significance (*P* < .05): sex, preoperative skin temperature, intraoperative NRS score, immediate postoperative HDSS score, preoperative HADS score, and surgical segment (overall comparison between thoracic and lumbar groups). Detailed statistical results of the univariate analysis are presented in Table 3.

**Table 3:** Univariate and multivariable analysis for CH.

Variables		CH			
		Univariate analysis		Multivariable analysis	
		OR (95% CI)	*P* value	OR (95% CI)	*P* value
Sex		1.54 (1.01, 2.35)	.044	1.69 (1.08, 2.68)	.022
Female Male	N = 131 (63.9%) N = 150 (73.2%)				
BMI (kg/m^2^)	21.78 (19.57–24.23)	1.06 (0.99, 1.13)	.112		
Family history		1.27 (0.81, 2.02)	.303		
No Yes	278 (67.8%) 132 (32.2%)				
Duration	17.00 (12.00–23.00)	1.01 (0.98, 1.03)	.659		
Total area (%)	11.00 (6.00–12.00)	0.98 (0.95, 1.02)	.329		
Smoking		1.78 (0.97, 3.46)	.075		
No Yes	346 (84.4%) 64 (15.6%)				
Preoperative PI	1.34 (0.89–1.96)	1.21 (0.99, 1.50)	.069		
Postoperative PI	7.73 (5.85–10.28)	1.06 (1.00, 1.13)	.062		
Preoperative T (°C)	30.27 (28.90–31.80)	1.13 (1.02, 1.26)	.022	1.12 (1.00, 1.25)	.051
Postoperative T (°C)	34.50 (33.64–35.15)	1.13 (0.97, 1.32)	.115		
NRS	6 (4–7)	1.17 (1.06, 1.30)	.003	1.16 (1.04, 1.29)	.008
Pre-HDSS	4 (4–4)	1.43 (0.88, 2.30)	.141		
T0-HDSS	1 (1–1)	0.55 (0.39, 0.75)	< .001	0.48 (0.33, 0.68)	< .001
Pre-DLQI	12 (9–13)	1.04 (0.97, 1.12)	.275		
Pre-HADS	11 (9–13)	1.11 (1.03, 1.20)	.007	1.11 (1.03, 1.21)	.007
Segment					
Thoracic vertebra	398 (97.1%)	0.22 (0.06, 0.71)	.014	0.32 (0.08, 1.16)	.095
subsegment	Ref: T3				
T3 (Ref)	89 (21.7%)	1		1	
T4	276 (67.3%)	0.92 (0.62, 1.37)	.685	0.93 (0.62, 1.40)	.712
T5	33 (8%)	0.80 (0.43, 1.49)	.475	0.80 (0.43, 1.49)	.478
Lumbar vertebra	12 (2.9%)	Ref: Thoracic vertebra			
subsegment	Ref: T3				
L3	12 (2.9%)	0.76 (0.29, 2.01)	.576	0.73 (0.27, 1.97)	.534

The values are presented as median (q1–q3)/n (%)/OR (95% CI). Significance set at *P* < .05.

BMI = body mass index, CH = compensatory hyperhidrosis, CI = confidence intervals, N/n = number of patients, NRS = numerical rating scale, OR = odds ratio, PI = perfusion index, Pre-DLQI = preoperative dermatology life quality index, Pre-HADS = preoperative hospital anxiety and depression scale, Pre-HDSS = preoperative hyperhidrosis disease severity scale, Ref = reference group, T = skin temperature of hyperhidrotic areas, T0-HDSS = immediate postoperative hyperhidrosis disease severity scale.

Subsequent hierarchical analysis of surgical segments was conducted with the T3 segment as the reference group. No significant differences in CH risk were observed among the T4, T5, and L3 segments compared with the T3 reference (all *P* > .05, Table 3), indicating that the surgical segment within the study range was not associated with CH occurrence in this cohort.

The 6 candidate variables screened by univariate analysis were further included in multivariate logistic regression analysis. Four variables were finally identified as independent predictors of postoperative CH with statistical significance (*P* < .05): male gender, higher intraoperative NRS score, lower immediate postoperative HDSS score, and higher preoperative HADS score. Neither surgical segment nor preoperative skin temperature reached statistical significance in the multivariate model (both *P* > .05) and thus were not included in the final predictive model (Table 3).

To enhance the clinical interpretability of our model, we converted the adjusted odds ratios of the 4 independent CH predictors from the multivariable model into absolute risks, based on the overall 68.5% baseline CH incidence over a median 23-month follow-up: male gender conferred an absolute CH risk of 78.6% (10.1% increase from baseline); each 1-point increase in intraoperative NRS score was associated with a 3.1-percentage-point rise in CH risk (range: 68.5% at NRS = 0 to 90.5% at NRS = 10); each 1-point increase in immediate postoperative HDSS (protective factor, odds ratio = 0.48) reduced CH risk by 17.4 percentage points (range: 68.5% at HDSS = 1 to 19.4% at HDSS = 4); and each 1-point increase in preoperative HADS score corresponded to a 2.2-percentage-point increase in CH risk (range: 68.5% at HADS = 0 to 94.6% at HADS = 21). These estimates provide an intuitive reference for clinicians to stratify CH risk in patients undergoing CT-guided RFS.

### 3.4. Dynamic nomogram model for predicting CH

**Figure 5.**
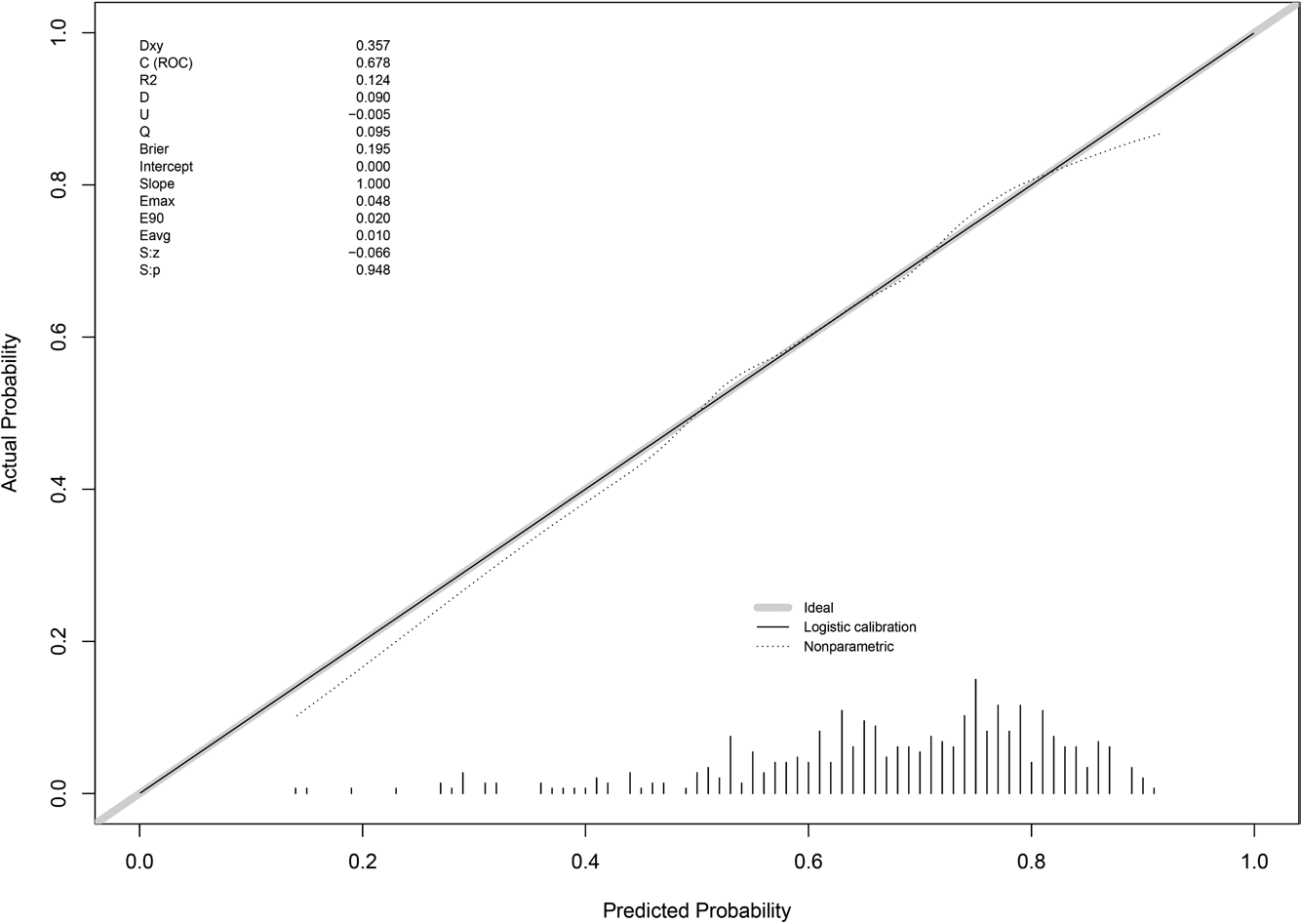
The dynamic nomogram for prediction of CH. The plot displays probability (with 95% confidence interval) of CH for patients after RFS. The actual explanatory values and their corresponding predictions are given in the “Numerical Summary” tab. CH = compensatory hyperhidrosis, RFS = radiofrequency sympathectomy.

Using the 4 independent predictors of CH confirmed by multivariable analysis, we developed and constructed a Shiny-based dynamic nomogram for individualized postoperative CH risk prediction (Fig. 5). This updated real-time dynamic nomogram realizes automatic risk calculation through 2 operation modes: direct value input and sliding bar adjustment. The free online calculator and nomogram visualization are fully open to clinicians and patients via the webpage (https://nomogram-ch.shinyapps.io/CHNomapp/).

**Figure 6.**
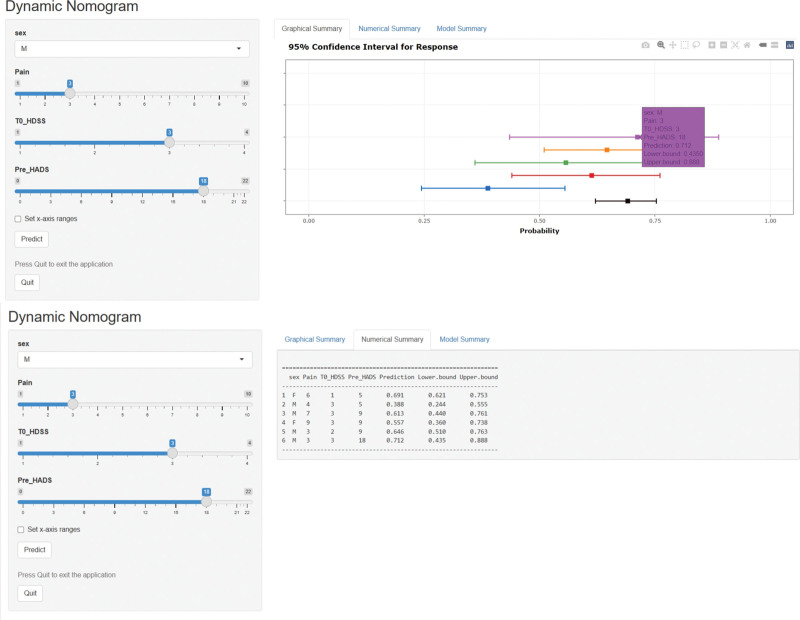
Calibration curves for CH prediction nomogram. The x-axis represents the predicted CH risk. The y-axis represents actual diagnoses of CH. The diagonal line represents a perfect prediction by an ideal model. The dotted line represents the performance of the nomogram, where a closer fit to the diagonal line represents a better prediction. CH = compensatory hyperhidrosis, Pre-HDSS = preoperative hyperhidrosis disease severity scale, T0-HDSS = immediate postoperative hyperhidrosis disease severity scale.

The model was internally validated by 100 bootstrap resamples, with a concordance index of 0.678 indicating moderate discriminative performance. In addition, the calibration curve (Fig. 6) showed a high degree of agreement between the model-predicted results and the actual clinical outcomes, confirming good consistency and reliability of the nomogram.

### 3.5. Treatment outcomes of patients

The median follow-up time of the entire cohort was 23 months (interquartile range: 12–36). The overall recurrence rate of PH was 23.9% at the last follow-up, with a significantly higher recurrence rate in the non-CH group than in the CH group (*P* = .001, Table 4). However, there was no statistically significant difference in patient satisfaction between the 2 groups (*P* = .689, Table 4).

**Table 4:** Postoperative experiences of patients who underwent RFS for primary hyperhidrosis.

	Total (N = 410)	Non-CH Group (N = 129)	CH Group (N = 281)	*P* value
Immediate results				
NRS	6 (4–7)	5 (4–7)	6 (5–8)	.002
Long-term result				
HADS–D	1 (0–2)	0 (0–1)	1 (0–2)	.010
DLQI–D	5 (2–8)	5 (2–9)	6 (2–8)	.411
Patient satisfaction	4 (3–4)	4 (3–4)	4 (3–4)	.689
Recurrence				.001
No	312 (76.1)	85 (65.9)	227 (80.8)	
Yes	98 (23.9)	44 (34.1)	54 (19.2)	
Operation to survey time (mo)	23 (12–36)	25 (12–37)	22 (11–36)	.102

The values are presented as median (q1–q3). Significance set at *P* < .05.

CH = compensatory hyperhidrosis, DLQI–D = delta DLQI (preoperative DLQI–postoperative DLQI), HADS–D = delta DLQI (preoperative HADS–postoperative HADS), mo = months, N = number of patients, NRS = numerical rating scale, RFS = radiofrequency sympathectomy.

## 4. Discussion

This retrospective observational study aimed to investigate the incidence, severity, distribution, temporal changes, and risk factors of CH following RFS in patients with PH. In a cohort of 410 patients with complete follow-up, multivariate logistic regression analysis identified male gender, higher intraoperative NRS scores, lower immediate postoperative HDSS scores, and higher preoperative HADS scores as independent risk factors for CH. A dynamic web-based nomogram was subsequently developed for individualized risk prediction.

RFS is a novel treatment option for patients with moderate-to-severe PH. Previous studies have focused on exploring the efficacy of this procedure, but related studies have highlighted that a considerable number of patients still experience CH after procedure, impacting overall patient satisfaction. This study showed patient experiences with RFS at our institution. Based on regular telephone follow-ups regarding CH, we observed post-RFS problems and analyzed the risk factors for CH.

Previous studies have shown that CH occurred with both interventions, with endoscopic thoracic sympathectomy (ETS) showing a greater likelihood and more severe degree of CH, despite its significantly improved effectiveness.^[14]^ Our findings confirm that CH is a universal postoperative complication of sympathetic nerve intervention for PH, regardless of the surgical modality. Further extend existing evidence by showing that the vast majority of RFS-related CH was well-tolerated by patients, with only a very small proportion of individuals reporting complete intolerance. This clinically relevant finding supports CT-guided RFS as a safe, minimally invasive alternative to ETS for patients with PH, particularly for those with high preoperative concerns about severe, debilitating postoperative CH.

Most existing research on CH risk factors has focused on ETS cohorts, with landmark studies like Chang et al^[15]^ establishing that more caudal sympathectomy levels correlate with lower CH incidence/severity (rooted in the sympathetic trunk’s rostral-to-caudal anatomy, where higher-segment ablation causes broader denervation) and Hong et al^[16]^ validating that multi-segment ETS increases severe CH risk versus single-segment resection; yet significant heterogeneity in surgical modalities limits the generalizability of these ETS-derived principles, as supported by RFS studies showing single-segment low-level (T4/L3) RFS yields substantially lower CH rates (7.96–53.3%) than high-segment ETS.^[5,14,17,18]^ In contrast, our stratified analysis of a CT-guided RFS cohort found no significant CH risk differences across T3-T5 and L3 segments (T3 as reference), which we attribute to 3 evidence-backed factors: exclusion of the high-risk T2 segment, small lumbar sample size limiting statistical power, and RFS’s precise focal single-segment thermal ablation (distinct from ETS’s wide nerve transection [including multi-segment resection]) that minimizes inter-segment denervation variability. This underscores that ETS-based CH risk principles cannot be directly extrapolated to RFS, guiding clinical decision-making to prioritize primary symptom control over segment-specific CH risk when selecting T3-T5/L3 for RFS.

Epidemiological survey found that the incidence of PH varied according to sex (females > males; 2.29% vs 1.94%), geographical distribution (southern and coastal > inland; 2.81% vs 1.53%), age (peak incidence of hyperhidrosis, 7–15 years), and genetic differences (25.40% had a family history),^[19]^ therefore, we included these variables. It indicates that gender is associated with the incidence of CH and is one of its risk factors. Notably, males have a higher likelihood of developing CH, with 150 cases accounting for 73.2% of male patients, which may be related to hormone levels, although further exploration of the specific mechanism is warranted.

To clarify the temporal dynamics and mechanisms of CH after RFS, we serially assessed CH severity in patients with PH. We found that nearly 50% of patients developed CH within 2 weeks and over 63% by 3 months postoperatively, consistent with previous studies.^[17]^ Many patients reported immediate nontarget area sweating before discharge, a phenomenon explained by the hypothalamic thermoregulatory reflex mechanism^[7]^ (where primary site denervation triggers compensatory homeostasis) but often masked by environmental, emotional, or dietary factors.^[17]^ While our data show CH severity stabilizes once established, this contrasts with reports of spontaneous resolution in some cohorts,^[17]^ likely due to RFS’s focal thermal ablation (vs ETS’s broad nerve transection) limiting denervation scope^[17,18]^ underscoring the need for ≥ 6-month follow-up to capture full CH outcomes.

This study verified that the occurrence of CH was not related to the number of hyperhidrosis sites (i.e., the size of the area) because the segments of radiofrequency thermocoagulation were based only on the main sites that troubled the patients. Previous studies have demonstrated a close association between negative emotions such as anxiety and depression with the occurrence or severity of hyperhidrosis.^[20,21]^ One study suggests that anxiety and depression are more common in patients with PH, especially generalized PH or facial PH, than in those without PH, which was a positive correlation between the severity of PH and the prevalence of anxiety and depression.^[20]^ However, whether these emotions are linked to the development of CH remains unclear. We assessed patients’ psychological status using the HADS before the procedure. In this study, the results showed that higher pre-HADS scores are a risk factor for CH, which may be related to preoperative education. Therefore, it is essential to reduce patients’ anxiety levels before surgery. The highest proportion of preoperative DLQI scores was in the range of 11 to 20, indicating that sweating had a huge impact on life, socialization, and work. However, a high preoperative DLQI score did not correlate with the incidence of CH.

This study had several limitations. First, as a retrospective study, there is inevitable recall bias and subjective bias, although we tried to minimize the recall bias for each patient by consulting the case data. Second, the performance of the constructed model in predicting CH was not superior, which may be related to an insufficient sample size. In the future, it will be necessary to expand the sample size and perform external verification to improve the generalizability of the model. To improve efficiency and reduce the incidence of postoperative CH, incision, entrapment, and intercostal nerve reconstruction techniques are constantly being explored.^[22,23]^ Meanwhile, this study did not include higher thoracic segments such as T2, and the sample size of the lumbar segment was relatively small, which may have limited the detection of the segmental effect.

This study’s strengths include being one of the largest single-center cohorts focused on CT-guided RFS for PH, with systematic long-term CH tracking via standardized tools, a dedicated CH database enabling robust identification of independent risk factors (sex, NRS score, immediate postoperative-HDSS, pre-HADS score) and development of the publicly accessible web-based dynamic nomogram for individualized CH risk prediction, strict adherence to Strengthening the Reporting of Observational Studies in Epidemiology guidelines with rigorous bootstrap validation and bias mitigation, and novel RFS-specific insights into CH dynamics and segment effect heterogeneity that complement ETS-dominated literature; future research should focus on multi-center prospective external validation of the nomogram, expanding surgical segments (e.g., T2, multi-segment) and lumbar RFS sample size to clarify ablation level/range-CH risk relationships, basic/translational studies on the mechanisms of identified risk factors, prospective interventional trials of targeted strategies for high-risk populations, and long-term (≥ 2 years) follow-up to assess CH durability, nomogram performance, and quality of life outcomes.

## 5. Conclusion

Male gender, higher NRS, lower immediate postoperative HDSS, and higher preoperative HADS are significant risk factors for CH following RFS. The nomogram serves as a useful predictive tool for this condition. This will allow patients to make informed decisions about personalized treatment options. Future research should focus on multi-center external validation of the nomogram and mechanistic exploration of identified risk factors to optimize personalized perioperative management of CH.

## Acknowledgments

The author would like to express gratitude to all the doctors in the Pain Department of Jiaxing University Affiliated Hospital for their support and assistance in this research.

## Author contributions

**Conceptualization:** Chaobo Ni.

**Methodology:** Chaobo Ni, Bohan Hua.

**Writing – original draft:** Chaobo Ni.

**Data curation:** Zixin Han.

**Resources:** Huadong Ni.

**Writing – review & editing:** Huadong Ni, Ming Yao.

**Supervision:** Ming Yao.
